# Distribution of different plant life forms on tropical islands: patterns and underlying mechanisms

**DOI:** 10.3389/fpls.2025.1566156

**Published:** 2025-04-02

**Authors:** Chengfeng Yang, Jingyan Zhao, Shengzhuo Huang, Shurong Zhou, Yikang Cheng

**Affiliations:** ^1^ Key Laboratory of Genetics and Germplasm Innovation of Tropical Special Forest Trees and Ornamental Plants, Ministry of Education, School of Tropical Agriculture and Forestry, Hainan University, Haikou, China; ^2^ Hainan Key Laboratory for Research and Development of Natural Products from Li Folk Medicine, Institute of Tropical Bioscience and Biotechnology, Chinese Academy of Agricultural Sciences, Haikou, China; ^3^ School of Ecology, Hainan University, Haikou, China

**Keywords:** ecological strategies, plants life forms, island area, island biogeography, island isolation, species abundance, species richness

## Abstract

**Introduction:**

Island biogeography theory posits that both island area and isolation significantly influence species distribution patterns and community structure. This study investigates the effects of island area and isolation on plant community structure, specifically focusing on the variation in species richness and abundance among different plant life forms (i.e., trees and shrubs) on tropical islands in the South China Sea.

**Methods:**

We surveyed woody plants and collected soil samples from 20 tropical islands in the South China Sea, analyzing how island area, isolation, climate and soil factors influence plant communities across different life forms (trees vs. shrubs).

**Results:**

The results indicate that species richness increases with island area and decreases with isolation, which aligns with the classic predictions of island biogeography. However, plant abundance exhibits a more complex pattern: tree abundance is positively correlated with island area and negatively correlated with isolation, while shrub abundance shows the opposite trend. Furthermore, the relative tree richness and abundance are predominant on larger, less isolated islands, whereas shrubs are more prevalent on smaller, more remote islands. These contrasting patterns suggest that different life forms adopt distinct ecological strategies within island ecosystems. The structural equation model (SEM) revealed that island area, isolation, and climatic factors directly affect the richness and abundance of trees but not shrubs. Additionally, the indirect effect of soil pH has proven to be a crucial environmental factor in shaping plant community structure.

**Discussion:**

Overall, this study highlights the multifaceted roles of geographic, climatic, and soil factors in determining the composition of island plant communities across different life forms. The findings have important implications for island conservation, as they provide a deeper understanding of how plant communities respond to spatial and environmental factors, aiding in the management of biodiversity on tropical islands.

## 1 Introduction

Island biogeography theory plays a critical role in ecology, with its core principle being the positive correlation between area and species richness, as well as the negative correlation between isolation and species richness (MacArthur and Wilson, 1967). Island ecosystems, serve as “natural laboratories” for ecological research, providing unique conditions that enable scientists to investigate the mechanisms of species formation, extinction, and ecological adaptation, thereby enhancing our understanding of biodiversity maintenance and the dynamic processes of species distribution (Whittaker and Fernández-Palacios, 2007; Losos and Ricklefs, 2009; Whittaker et al., 2017). In the context of global change and increasing human disturbance, the significance of island studies have become even more pronounced (Fernández-Palacios et al., 2021; Manes et al., 2021).

Current research on island biogeography theory primarily focuses on the relationship between species richness and island area or isolation. However, community composition, including both species and functional diversity, is also highly sensitive to changes in island area, isolation, and environmental factors, which further influence ecosystem functions (MacArthur and Wilson, 1967; Warren et al., 2015; Spaak et al., 2017; Losos and Schluter, 2000; Schrader et al., 2020; Li X. et al., 2024). For instance, studies on plant abundance in contemporary ecological research are limited, yet abundance plays a crucial role in ecosystem functions, and its trends may not necessarily align with those of species richness (Ehrlén and Morris, 2015). A study on ecosystem services found that the abundance of common species is a major driver of ecosystem service functions, while species richness is not significantly correlated (Winfree et al., 2015). Additionally, a meta-analysis revealed that nitrogen enrichment reduced both plant species richness and abundance; however, the magnitude of the response varied due to differences in biodiversity indicators, environmental factors, and experimental contexts (Midolo et al., 2019).

Compared to mainland ecosystems, island ecosystems typically face more limited resources and harsher environmental conditions, which drive plants to develop unique and efficient adaptive strategies to cope with these challenges (Craine et al., 2012; Volaire, 2018). For example, in arid regions, short-lived plants fully exploit the periods each year when temperature and precipitation are moderately favorable for growth, completing their entire life cycle within this window and thus adopting a shortened life history strategy (Li et al., 2019; Tao et al., 2022). In island environments, the survival and reproduction of trees and shrubs necessitate more efficient resource utilization, which may, in turn, influence their distribution and diversity within the island ecosystem (Han et al., 2020). Trees are taller with larger root systems, enabling efficient resource capture and ecological stability. However, in relatively harsh island environments, their distribution is constrained by competition and growth conditions, making them more likely to concentrate in more favorable habitats (Shirima et al., 2016; Ziegler et al., 2024). In contrast, shrubs, with their shorter heights and dense branching structures, demonstrate greater adaptability and can achieve higher individual densities in competitive environments, thereby occupying more ecological niches and forming rich vegetation layers (Wilson, 1995; Götmark et al., 2016). Due to differing survival strategies and adaptations, plants with various life forms may respond differently to island area, isolation, and environmental factors (Xie et al., 2024). For instance, a study along an altitudinal gradient in the eastern Himalayas of Nepal found that plants of different life forms exhibited distinct relationships with various environmental factors (Bhattarai and Vetaas, 2003). Additionally, the relative abundance and density of different life forms within a community are crucial. The survival strategies and environmental responses of trees and shrubs differ, and their relative abundance and density can reveal their interrelationships and adaptation strategies within the ecosystem, the relative proportion of trees and shrubs reflects different successional stages of island vegetation and, to some extent, the stability of the island ecosystem (Morales et al., 2012; Yang et al., 2020). Some studies have shown that shrubs facilitate tree establishment in peat bogs, and once a certain number of trees are established, they further enhance shrub growth. The varying proportions of trees and shrubs within a community can have significant effects on its health and stability (Holmgren et al., 2015). Different relative proportions of trees and shrubs influence both species diversity and ecosystem function. Shrub dominance may aid soil restoration and expand habitat occupancy, while tree dominance enhances community stability and productivity (Lavorel and Garnier, 2002; Zizka et al., 2014). These ecological functions are vital for island ecosystem conservation, particularly through optimizing vegetation structure and fostering species interactions to improve ecosystem sustainability.

When exploring the relationship between species richness and island area, several mechanisms have been proposed, including the “passive sampling effect”, which suggests that island size may influence species numbers primarily through limitations on sample space rather than ecological factors (Connor and McCoy, 1979); the “habitat diversity effect”, which posits that species richness on islands is closely related to the diversity of habitat types and structures (Bracewell et al., 2018; Zhou et al., 2024); and “ecological drift”, which implies that changes in species populations may result from random factors rather than selective pressures (Gilbert and Levine, 2017; Leibold and Chase, 2018). Numerous studies have demonstrated that these mechanisms play varying roles across different island systems (Li S. et al., 2024; Zhou et al., 2024). However, these mechanisms may overlook additional factors that could further explain island plant community composition, such as climatic conditions and habitat quality (e.g., soil nutrients, soil pH), which also influence island plant communities (Wu et al., 2022; Yuan et al., 2024; Zhou et al., 2024). A substantial body of evidence indicates that climate significantly affects plant growth, reproduction, and distribution (Cabral et al., 2014; Ramirez et al., 2020). Furthermore, soil properties, particularly soil nutrients and pH, exert distinct effects on plants, which vary across different ecosystems (Barker and Pilbeam, 2015; Barrow and Hartemink, 2023; Duan et al., 2023). By investigating the effects of island area, isolation, and climate, we can better understand how these factors influence island plant species richness and the abundance of various plant life forms. This occurs directly or indirectly through soil characteristics, providing a more comprehensive understanding of the dynamic changes in island ecosystems and their responses to external pressures. This knowledge provides essential scientific insights for ecological conservation and resource management.

Due to the diverse adaptive strategies exhibited by different life forms, this study focuses on examining the varying responses of trees and shrubs to island area, isolation, and other environmental factors on the islands of the South China Sea. Specifically, the objectives of this study are to: 1) explore the relationships between plant species richness and the abundance of different life forms (trees and shrubs) in relation to island area and isolation; 2) assess how the relative abundance and relative density of trees and shrubs change with variations in island area and isolation; and 3) identify the key environmental and ecological factors that influence the richness, abundance, and relative distribution of trees and shrubs across different islands. Based on island biogeography theory, we hypothesize that larger and less isolated islands will have higher tree abundance, while smaller and more isolated islands will favor shrubs due to their superior adaptability and more efficient reproductive strategies. These insights will help clarify the mechanisms behind the composition of island plant communities and contribute to better understanding of the ecological dynamics at play in these unique environments.

## 2 Materials and methods

### 2.1 Study site and sampling method

As an important tropical marine ecosystem, the South China Sea is home to numerous islands, coral reefs, and shallows, exhibiting rich biodiversity and complex marine ecological environments. This region not only provides habitats and breeding grounds for a variety of marine organisms but also creates conditions conducive to the growth of diverse tropical and subtropical plant species. Despite the abundance of islands in this area, some are too small or lack sufficient vegetation to meet the criteria for plant surveys. Consequently, we selected 20 representative islands with woody vegetation that have experienced minimal disturbance for investigation from April 2023 to May 2024.

For each island, we established 20 m × 20 m permanent sampling plots, with the number of plots proportional to the logarithmic transformation of the island area (log10) (**Supplementary Figure S1**). Additionally, we took topography into account when selecting the specific locations of the plots, ensuring that the plot locations on the investigated islands covered all terrain types and different altitudes of the islands as much as possible. Additionally, we considered the vegetation conditions and habitat differences of the islands as supplementary information for plot selection. As a result, a total of 87 permanent plots were established across the 20 islands. To minimize the effects of spatial autocorrelation, we ensured that the distance between any two adjacent plots within each island exceeded 100 meters. For the smallest islands, while it was not entirely feasible to meet this standard, the distance between plots was maintained at over 40 meters.

On all selected islands, we conducted plant surveys and soil sampling within the established permanent plots. First, we utilized Real-Time Kinematic (RTK) positioning technology, provided by Xunwei Positioning, to accurately locate the four corners of each plot. We marked these positions by inserting white rubber tubes into the soil and enclosing the 20 m × 20 m plot with red plastic string. Within the marked plots, we surveyed all woody plants with a diameter at breast height (DBH) ≥ 1 cm, recording species names through morphological identification and subsequently calibrated the Chinese and Latin names of the species based on the “Flora of China” (Flora of Chinese, 2019).

To collect soil samples, we established three evenly distributed 2 m × 2 m quadrats along the diagonal of each plot, ensuring that the quadrats covered different microhabitats within the plot. In each quadrat, we randomly collected four soil samples to a depth of 0-10 cm from various locations within the quadrat. The soil samples from each quadrat were then combined and thoroughly mixed to create a composite sample for each quadrat. In total, 261 composite soil samples were obtained across all islands, corresponding to 87 plots and three quadrats per plot. For each soil sample, large particles were removed using a 2 mm sieve, and the samples were and stored at 4°C for subsequent measurements of their physicochemical properties.

### 2.2 Data acquisition

We classified these plants into tree or shrub categories based on their life forms, referencing the “Flora of China” (Flora of Chinese, 2019). Subsequently, we utilized the “vegan” package in R to calculate the species richness and abundance of all species, as well as those of trees and shrubs, respectively. Additionally, we assessed the relative richness and relative abundance of trees and shrubs for each plot.

We measured five physicochemical properties of soil from the collected samples that could potentially influence plant communities, using the following specific methods: Soil pH was determined with a Metter- S210 SevenCompact pH analyzer, employing a soil-to-water ratio of 1:2.5. Soil organic carbon (SOC) content was assessed using the K2Cr2O7 oxidation-reduction titration technique. Total nitrogen (TN) levels in the soil were quantified utilizing the semi-micro Kjeldahl method. Total phosphorus (TP) concentrations were measured through colorimetric analysis with a UV-visible spectrophotometer. Finally, total potassium (TK) content was extracted using 1 M ammonium acetate and analyzed via inductively coupled plasma technology.

Climate data were obtained from WorldClim’s global climate and weather database (Fick and Hijmans, 2017), specifically the WorldClim 2.1 dataset for the period 1970-2000. This version was released in January 2020. Annual mean temperature and annual precipitation data were extracted from the bioclimatic variables bio1 and bio12 in the historical climate dataset. Wind speed data were sourced from a dataset with a spatial resolution of 2.5 arc minutes. We processed the downloaded TIFF files using the “terra” package in R to extract annual mean temperature, annual precipitation, and annual mean wind speed data for the 20 surveyed islands.

### 2.3 Statistic analysis

In this study, we employed linear regression to assess the effects of island area and isolation on plant community structure. We used logarithmically transformed island area and isolation as independent variables to analyze their linear relationships with species richness and abundance, including total species, trees, and shrubs, as well as the relative richness and relative abundance of different plant life forms.

We applied principal component analysis (PCA) to simplify the data structure of climate and soil nutrient variables. For climate, we used annual mean temperature, annual precipitation, and annual mean wind. For soil nutrients, we included total nitrogen (TN), total phosphorus (TP), total potassium (TK), and soil organic carbon (SOC). The first principal component (PC1) from each PCA was retained as a composite predictor, as it explained the largest proportion of the variance-56.0% for climate variables and 54.7% for soil nutrients. In PC_climate_, annual precipitation (-0.711) contributed the most, while in PC_soil_, both nitrogen (-0.658) and soil organic carbon (-0.653) had the largest contributions. These first principal components were used as composite predictors for climate and soil variables in subsequent analyses (**Supplementary Table S1**).

We employed a piecewise structural equation model (SEM) to assess the direct effects of island area, isolation, and climate variables (PC_climate_) on total plant richness and abundance, as well as the absolute and relative richness and abundance of each life form. Additionally, we examined the indirect effects mediated by soil nutrients (PC_soil_) and soil pH. The psem function in the piecewiseSEM package (Lefcheck, 2016) was utilized for the SEM analysis. Specifically, this method performs confirmatory path analysis based on the results of directed separation tests (Shipley, 2009, 2013). Model fit was evaluated using goodness-of-fit statistics, and the best model was selected by comparing the Akaike Information Criterion corrected for small sample size (AICc) values between the full model and simplified models (Grace, 2006). The model with the lowest AICc value was designated as the final model for the study (Shipley, 2013). Furthermore, the overall model fit was assessed using Fisher’s C statistic (**Supplementary Table S2**). If the p-value of Fisher’s C statistic exceeded 0.05 (i.e., the null hypothesis was accepted), the model was considered to have a good fit (Shipley, 2009). All statistical analyses were conducted using R 4.4.1.

## 3 Results

### 3.1 Response of richness of different life forms to island area and isolation

**Figure 1:**
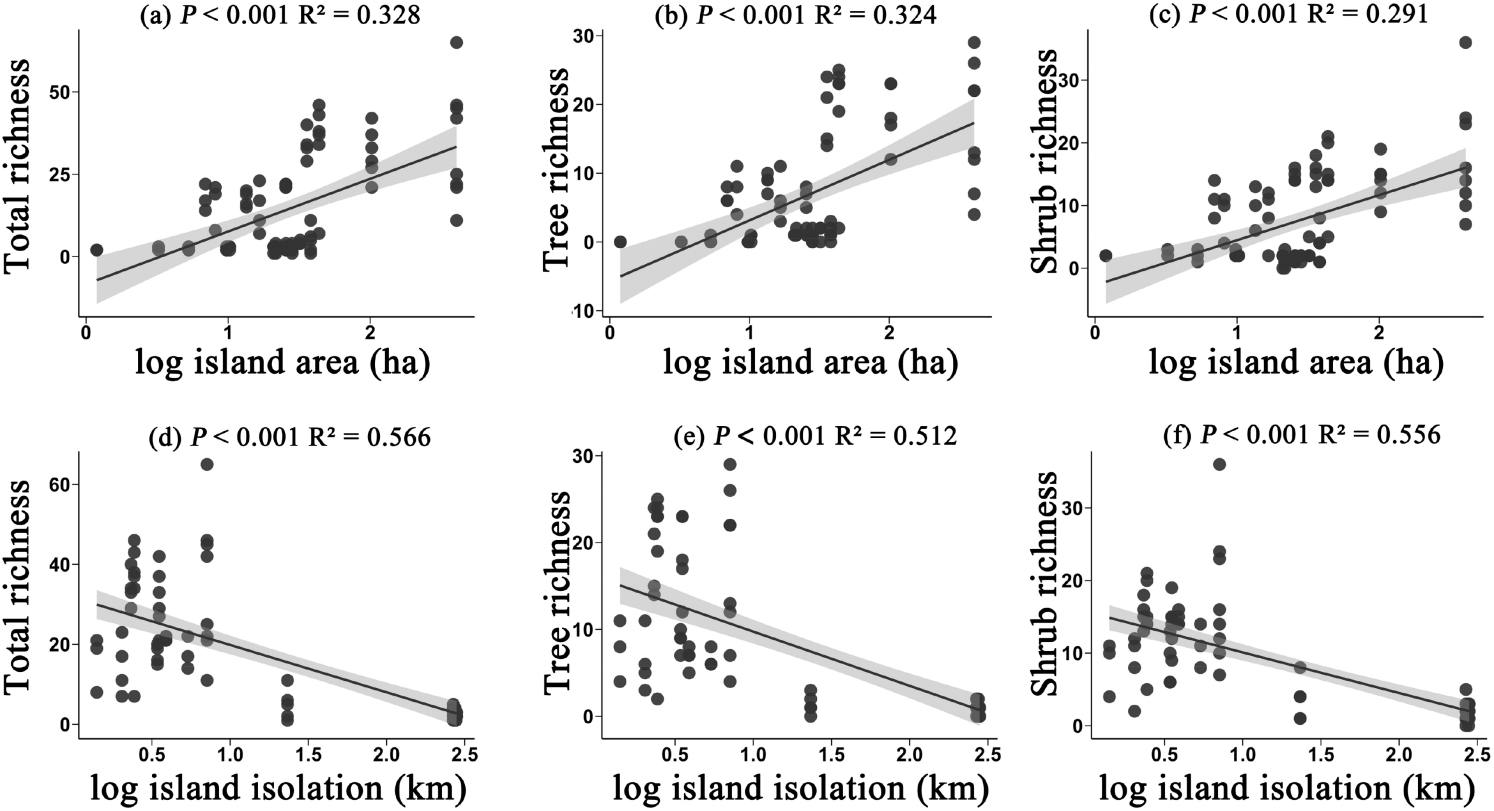
The effects of island area on the richness of all species **(a)**, tree richness **(b)**, and shrub richness **(c)**; and the effects of island isolation on the richness of all species **(d)**, tree richness **(e)**, and shrub richness **(f)**. Species richness refers to the taxonomic diversity within each plot, specifically the number of species present in a plot.

In terms of species richness, the richness of all species, trees, and shrubs exhibited similar trends in relation to island area and isolation. Specifically, with an increase in island area, the richness of all three groups significantly increased (*P* < 0.05) (**Figures 1a-c**). This phenomenon suggests that larger islands can provide more niches and resources, thereby supporting a greater number of species for survival and reproduction. Conversely, as isolation increased, the richness of all species, trees, and shrubs significantly decreased (*P* < 0.05) (**Figures 1d-f**). This trend may be related to the limitations on species dispersal abilities; more isolated islands often struggle to maintain high levels of species diversity. These results further emphasize the important influence of island area and isolation on plant community structure.

### 3.2 Response of abundance of different life forms to island area and isolation

**Figure 2:**
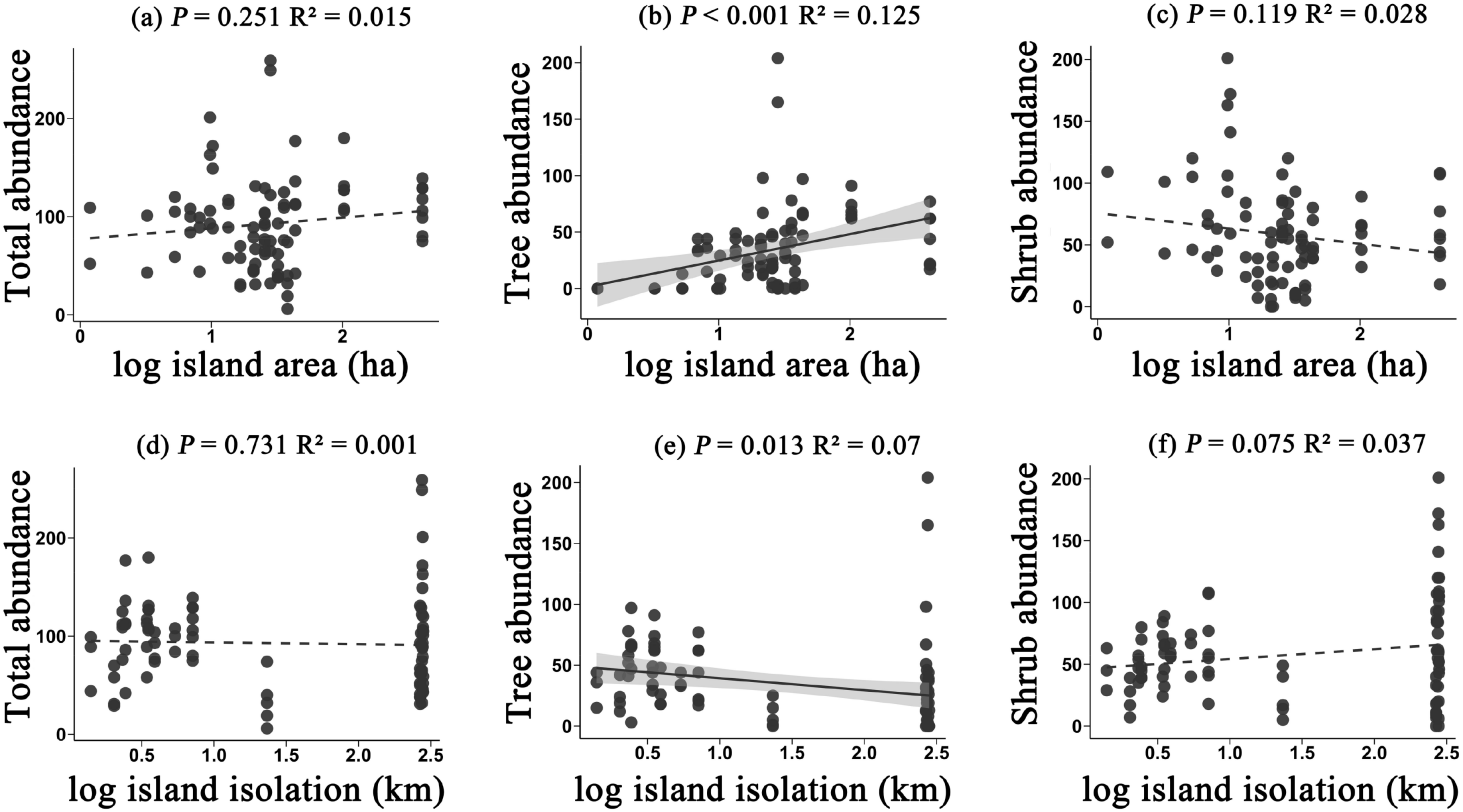
The effects of island area on the abundance of all species **(a)**, tree abundance **(b)**, and shrub abundance **(c)**; and the effects of island isolation on the abundance of all species **(d)**, tree abundance **(e)**, and shrub abundance **(f)**. Plant abundance refers to the number of individuals present in each plot.

In terms of species abundance, the relationships between the abundance of total species, trees, and shrubs with island area and isolation exhibited certain differences. Specifically, the abundance of total species did not show a significant relationship with either island area or isolation (*P* = 0.251, *P* = 0.731) (**Figure 2a, d**). However tree abundance increased significantly with island area, demonstrating a clear positive correlation (*P* < 0.001) (**Figure 2b**). Tree abundance, on the other hand, decreased with isolation (*P* = 0.013) (**Figure 2e**). In contrast, the trend for shrubs was opposite to that of trees. Shrub abundance showed a decreasing trend with island area (*P* = 0.119) (**Figure 2c**) but an increasing trend with isolation (*P* = 0.075) (**Figure 2f**), though the relationships were not statistically significant. These findings suggest that trees are more sensitive to the island area and isolation, possibly relying more on ecological spaces to maintain population stability. At the same time, the differing responses in abundance between trees and shrubs further reflect the diversity of adaptive strategies among different life forms in island environments.

### 3.3 Response of relative richness and relative abundance of different life forms to island area and isolation

**Figure 3:**
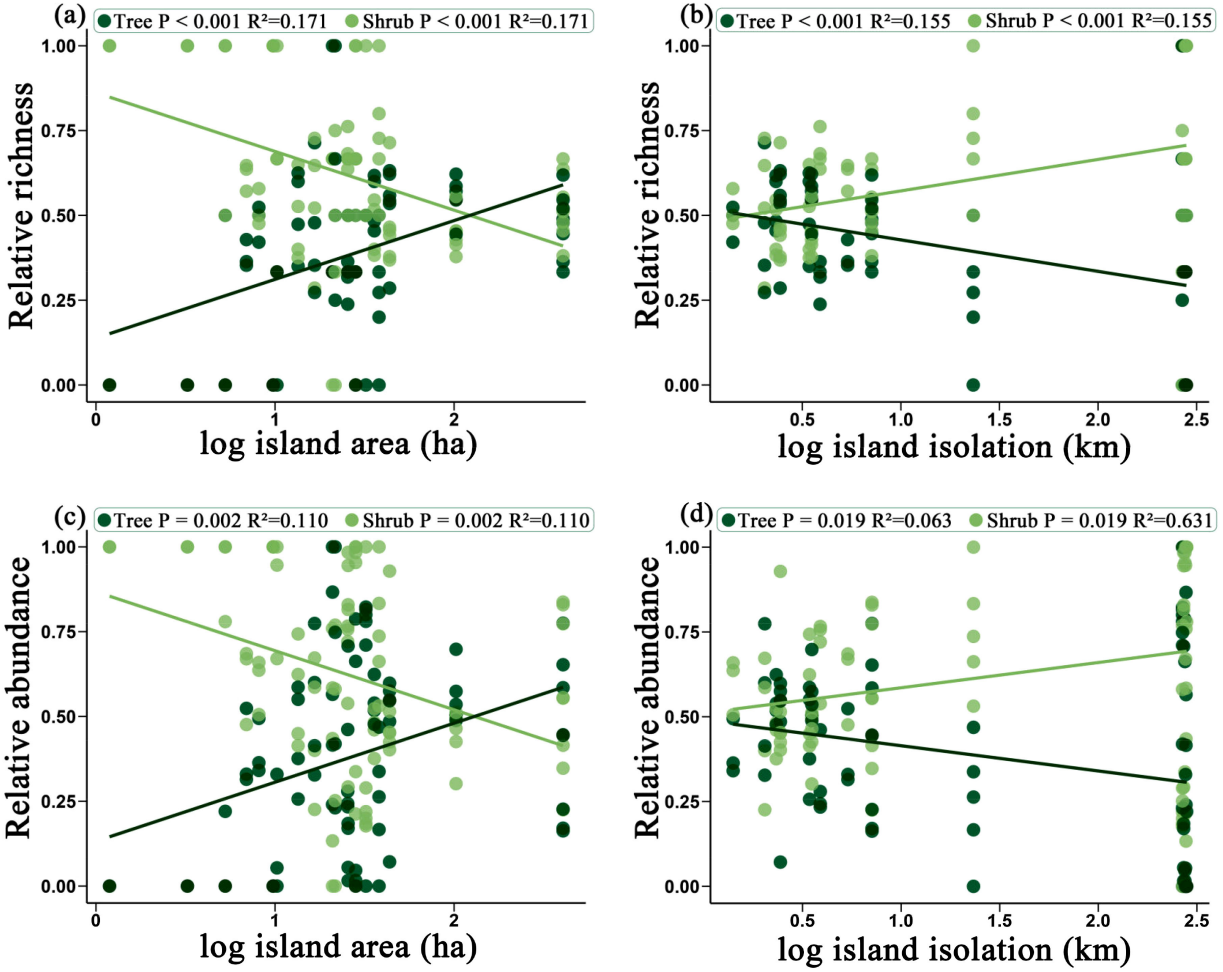
The effects of island area on the relative richness of trees and shrubs **(a)** and the relative abundance of trees and shrubs **(c)**; and the effects of island isolation on the relative richness of trees and shrubs **(b)** and the relative abundance of trees and shrubs **(d)**. Dark green represents trees, while light green represents shrubs.

As island area increases, both relative richness and relative abundance of trees significantly increased, while the relative richness and relative abundance of shrubs significantly decreased (*P* < 0.001, *P* = 0.002) (**Figure 3a, c**). This indicates that large island area favors the spread and colonization of trees, while reducing the relative dominance of shrubs. Conversely, as isolation increases, both the relative richness and relative abundance of trees significantly decreased, while the relative richness and relative abundance of shrubs significantly increased (*P* < 0.001, *P* = 0.019 (**Figure 3b, d**). This suggests that on highly isolated islands, shrubs have a higher relative proportion within the community. In comparison, trees are more severely constrained in highly isolated environments, leading to a decrease in their proportion within the community. In summary, island area and isolation have significant effects on the relative proportions of trees and shrubs in a plant community. An increase in island area favors the richness and abundance of trees, whereas increased isolation promotes the relative proportion of shrubs in the community.

### 3.4 Direct and indirect effects of island area, isolation, and climate on the absolute and relative plant richness and abundance

**Figure 4:**
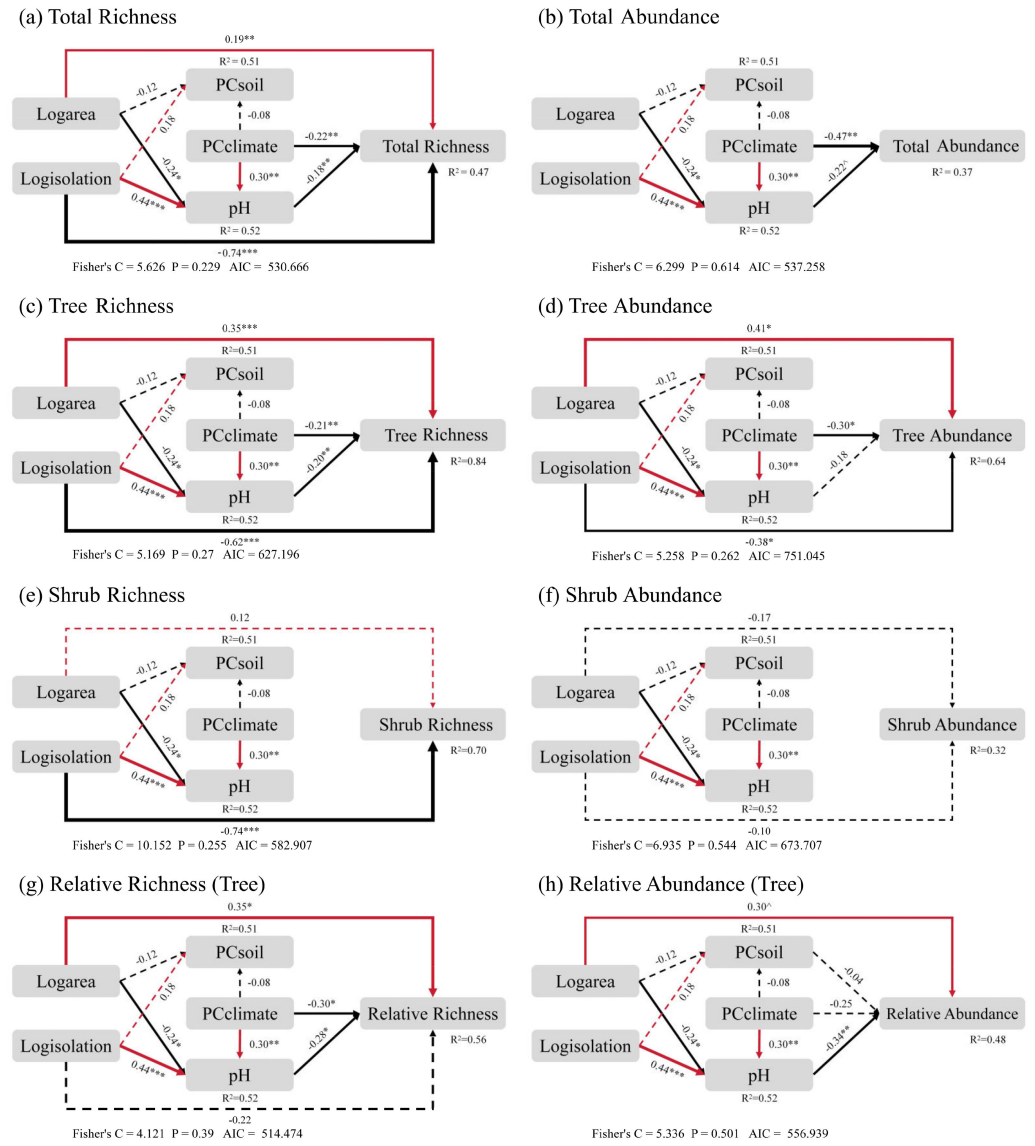
The final results of the Structural Equation Model (SEM) reveal the direct effects of island area, isolation, and climate on total species richness **(a)**, total species abundance **(b)**, tree species richness **(c)**, tree species abundance **(d)**, shrub species richness **(e)**, shrub species abundance **(f)**, relative richness of trees **(g)**, and relative abundance of trees **(h)**, as well as their indirect effects mediated by environmental factors such as soil nutrients and pH, respectively. Plant species richness was estimated based on the number of species observed in the plots, while abundance was estimated based on the number of individuals recorded. PC_climate_ represents the climate factors after dimensionality reduction through Principal Component Analysis (PCA), PC_soil_ refers to the soil nutrient factors after dimensionality reduction, and pH represents soil acidity. Red and black arrows are the standardized path coefficient variables, representing significant positive and negative pathways respectively, with the thickness of the line proportional to the strength of the path coefficient. R^2^ is the marginal value that represents the proportion of variance explained for each fixed variable in the model. The symbols denote statistical significance (***P < 0.001; **P < 0.01; *P < 0.05; ^*P* < 0.1).

The final structural equation model (SEM) revealed that the direct and indirect effects of island area, isolation and climate on the richness and abundance of different life forms varied. For all the species, island geographical characteristics and climate mainly exerted indirect effects on island plant richness and abundance through their influence on soil pH, rather than soil nutrients Island area, isolation, and climate factors also directly influenced species richness. Species abundance, on the other hand, was only directly influenced by climate factors, while island area and isolation had no direct effect on it (**Figure 4a, b**). For trees, the effects of island area, isolation, and climate on their richness followed a similar pattern to that of total species richness, but with different coefficients. Tree abundance, however, was directly influenced only by island area, isolation, and climate (**Figure 4c, d**). For shrubs, richness was mainly directly influenced by island isolation, while abundance was neither directly nor indirectly affected by affected by island geographical characteristics and climate (**Figure 4e, f**). Since the relative richness and relative abundance of trees and shrubs sum to 1, they exhibited a negative correlation. Therefore, the trends for trees were opposite to those of shrubs (**Supplementary Figures S2a, b**). For the relative richness of trees, island area, isolation, and climate factors influenced it via soil pH. The relative richness of trees also increased with island area. Island isolation did not have a direct effect on relative richness of trees (**Figure 4g**). For relative abundance, the impact of island area was more pronounced, while island isolation and climate factors only influenced it indirectly through soil pH and had no direct effects (**Figure 4h**).

## 4 Discussion

### 4.1 Effects of island area and isolation on plant richness and abundance

While traditional studies in island biogeography have mainly focused on the relationship between species richness and island area or isolation, our study further explores the differences in the responses of plant community abundance to island area and isolation, revealing significant ecological adaptations between trees and shrubs. Overall, plant species richness significantly increased with larger island areas, while it decreased markedly with greater isolation, a trend that aligns with the classic predictions of island biogeography (MacArthur and Wilson, 1967). This positive correlation between island area and species richness, as well as the negative correlation between isolation and species richness, was observed for the entire plant community and when categorized by life forms (Weigelt and Kreft, 2013; Portillo et al., 2019). These findings suggest that both island area and isolation play an important role in influencing plant species richness, and these patterns remain consistent regardless of the classification of different plant life forms (Kubota et al., 2015; Xie et al., 2024). However, the trends in plant abundance on these islands did not fully correspond with species richness. Notable differences in abundance trends were observed between trees and shrubs across various plant life forms. Specifically, the trend in tree abundance was consistent with that of its species richness; tree abundance significantly increased with island area but decreased significantly on highly isolated islands. This indicates that the growth and expansion of trees may depend on the ample resources and habitats available on larger islands, while high levels of isolation hinder seed dispersal and colonization, further limiting the increase in tree population density on isolated islands (Whittaker and Fernández-Palacios, 2007; Shirima et al., 2016; Nogales et al., 2024).

In contrast, the relationship between shrub abundance and island area and isolation exhibited an opposite trend. On islands with greater isolation, shrub abundance was higher, although this trend was close to being statistically significant. This phenomenon may be attributed to the relative reduction of tree abundance and the ecological strategy of shrubs, which makes their abundance insensitive to island area and isolation, as well as the formation of high-density monocultures. For instance, shrubs typically possess high vegetative reproduction capabilities and rapid growth rates; many shrubs on islands exhibit rhizomes and stolons, which enable them to occupy habitats with limited resources or restricted seed dispersal, thereby enhancing species persistence (Wilson, 1995; Götmark et al., 2016; Ottaviani et al., 2020). As observed in this study, islands such as Beishazhou Island and Zhongshazhou Island, which are characterized by higher isolation and smaller areas, displayed relatively low species richness. However, plant abundance was particularly high due to the dominance of a single species, i.e., *Scaevola taccada*. This suggests that while soil and climatic conditions on highly isolated islands may not be suitable for the growth of all plant types, larger islands may also suppress or inhibit the growth of certain plants through resource competition, with trees and shrubs showing different adaptive responses.

It is important to note that the differing patterns in species abundance and species richness suggest that the number of species and the abundance of individuals may be driven by distinct ecological mechanisms. Therefore, a comprehensive examination of these patterns should take into account various ecological processes from multiple perspectives within island ecosystems.

### 4.2 The regulation of the relative proportions of trees and shrubs by island area and isolation

This study revealed that island area and isolation significantly influence the relative richness and abundance of different plant life forms within the plant community. As island area increases, the relative richness and abundance of trees significantly increased, while the relative richness and abundance of shrubs significantly decline. This phenomenon aligns with the classical “area effect” in island biogeography, where larger islands provide more habitats and resources, thereby facilitating the expansion and survival of species (Connor and McCoy, 1979; Leibold and Chase, 2018; Li S. et al., 2024). For trees, larger islands not only offer more habitat but also maintain more stable environmental conditions, particularly in resource-rich environments where trees may dominate interspecific competition, driving their relative abundance and richness upward (Yan et al., 2023). In contrast, the relative richness and abundance of shrubs are lower on larger islands, which may be attributed to the expansion and competition of tree populations (Barnes and Archer, 1999). On larger islands, trees may dominate due to their spatial requirements for growth and their efficiency in resource utilization, which limits the expansion of shrubs (Grime, 2006). However, on smaller islands, the plant community may be more strongly influenced by edge effects, which favor the competition of shrubs in lower-quality habitats, resulting in a noticeable increase in their relative richness and abundance (Harper et al., 2005).

The increase in island isolation significantly reduced the relative richness and abundance of trees, while the relative richness and abundance of shrubs significantly increased. This is likely due to differences in dispersal mechanisms and colonization success rates. On smaller and more isolated islands, the difficulty of plant colonization increases. Shrubs, compared to trees, possess more flexible dispersal mechanisms and stronger reproductive and adaptive abilities to survive in harsh environments (Zizka et al., 2014; Ehmet et al., 2021). For instance, one of the most frequently occurring species in this survey, *Scaevola taccada*, has heteromorphic fruits—one type with softwood and fleshy pulp, and another with only pulp—that can be dispersed both by seawater and indirectly by birds, greatly enhancing the species’ dispersal efficiency (Emura et al., 2014). In cases where dispersal is challenging, shrubs may benefit relatively more from increased isolation, as their adaptive advantages are more pronounced in these environments (Gillespie et al., 2008). Shrub species, characterized by higher growth rates and the ability to rapidly respond to environmental changes while tolerating local resource limitations, may quickly occupy new habitats, form dominant communities, and potentially further restrict the territorial expansion of trees (Fukami, 2015). These findings suggest that classical theories in island biogeography still play a significant role in shaping plant communities. However, the distribution and dynamics of plant populations are not determined solely by the physical environment; their ecological strategies and interspecies competition also contribute to shaping these patterns.

### 4.3 The driving mechanisms of island plant communities: the synergistic effects of geography, climate, and soil factors

Our results clearly indicate that the direct effects of island area and isolation are the most significant factors influencing plant communities on islands. The findings from the structural equation modeling (SEM) demonstrate that the direct effects of island area and isolation are highly significant, which is consistent with studies conducted on various islands. However, the impact of island area and isolation on species richness and abundance, as well as on different plant life forms, exhibits some variability (Schrader et al., 2020; Lee et al., 2022; Xie et al., 2024; Zhou et al., 2024). Specifically, species richness is more readily influenced by island area and isolation, while the responses of trees and shrubs to these two factors differ. The survival strategies of trees indicate a greater reliance on the favorable living conditions provided by larger island areas (Connor and McCoy, 1979). Furthermore, the influence of area and isolation on plant communities extends beyond direct effects to include indirect effects as well.

We also found that climatic factors, such as temperature, precipitation, and wind speed, directly influence the community structure of plants on islands, particularly affecting trees (Harter et al., 2015; Irl et al., 2020). Compared to mainland environments, oceanic islands, influenced by surrounding seas, may have more favorable conditions in terms of precipitation and temperature. However, the isolation and smaller size of islands made their plant communities more sensitive to changes in these climatic factors. Therefore, in the fragile island environment, climatic factors can still directly impact plant growth, survival, and distribution (Cabral et al., 2014; Ramirez et al., 2020). Interestingly, precipitation contributed the most in the PC_climate_. However, trees and shrubs responded differently to precipitation. Structural equation modeling (SEM) revealed that trees were directly influenced by the precipitation-dominant PC_climate_, while shrubs were not. This is likely related to the more robust adaptive strategies of shrubs. As observed in our study, on islands dominated by shrubs (with higher relative abundance and relative dominance of shrubs), the leaf thickness of these shrub species was greater, and in some cases, even succulent-like (e.g., *Scaevola taccada*, *Tournefortia argentea*), further corroborating the distinct ecological strategies of trees and shrubs on islands (Aneja et al., 2025). Although wind speed had a lower contribution to the climate principal component than precipitation, it remains an important factor. In tropical islands, intermittent extreme wind events, such as tropical storms or typhoons, can significantly impact plant communities, leading to tree falls or vegetation loss (Uriarte et al., 2019; Bauman et al., 2022). These disturbances may disrupt existing community structures and provide opportunities for more adaptable plants, such as shrubs or herbaceous species, to expand, thereby altering the species composition of the community (Murphy and Metcalfe, 2016). We also found that the effects of these climatic factors are not only reflected at the physiological level of plants but also indirectly influence plant communities by altering the soil pH on islands. For instance, changes in precipitation and temperature can cause fluctuations in soil acidity, which in turn affect nutrient uptake and plant growth rates (Jesus et al., 2015; Pugnaire et al., 2019).

We also found that climatic factors, such as temperature, precipitation, and wind speed, directly affect the community structure of plants on islands (Harter et al., 2015; Irl et al., 2020). Islands are more fragile than mainland environments, and temperature and precipitation directly influence plant growth, survival, and distribution (Cabral et al., 2014; Ramirez et al., 2020). However, these effects are not only reflected at the physiological level of plants; they also indirectly influence plant communities by affecting soil conditions on the islands, including soil nutrients and pH (Jesus et al., 2015; Pugnaire et al., 2019). Wind speed, as a unique climatic factor on islands, also has a significant impact on plant communities. Strong winds can directly affect the growth and stability of tall plants, such as trees, and alter species dispersal patterns by spreading seeds and pollen (Kuparinen et al., 2009; Bullock et al., 2012). Particularly in tropical islands, intermittent extreme wind events, such as tropical storms or typhoons, can significantly impact plant communities, leading to tree falls or vegetation loss (Uriarte et al., 2019; Bauman et al., 2022). Such disturbances may disrupt existing community structures and create opportunities for highly adaptable plants, such as shrubs or herbaceous species, to expand, thereby altering the species composition of the community (Murphy and Metcalfe, 2016).

Our study confirmed the important role of soil pH in influencing island plant communities. This is supported by the results from structural equation modeling (SEM), which indicated that soil nutrients (such as nitrogen and organic carbon) did not have a significant direct impact on plant community structure. Instead, island area, isolation, and climatic factors affected community structure through their influence on soil pH. As a key environmental factor in island plant communities, soil pH profoundly affects plant growth and species diversity. It influences plant communities through various ecological mechanisms, not only by directly restricting plant growth but also by indirectly shaping community structure through alterations in interactions among plants, the environment, and other organisms. Soil pH affects the efficiency of nutrient absorption by plants, particularly the availability of essential elements (Kemmitt et al., 2006; Penn and Camberato, 2019; Barrow and Hartemink, 2023). Furthermore, soil pH influences the diversity and functional activity of rhizosphere microorganisms, which further impacts plant community composition through plant-microbe interactions (Husson, 2013; Duan et al., 2023). At the community level, in soils with extreme acidic or alkaline conditions, species with strong tolerance often dominate ecological niches, while pH-sensitive species may be excluded from the community (Barker and Pilbeam, 2015; Deinlein et al., 2014; Mann et al., 2023). This selective effect significantly alters the species composition of the community, particularly in isolated island environments, where regional variations in soil pH can increase the heterogeneity of plant communities across different islands, our study provides a new perspective for island ecology, emphasizing the critical role of soil as an ecological factor.

## 5 Conclusion

Although many traditional studies in island biogeography focus on the relationship between species diversity and island area or isolation, fewer investigations have refined the analysis of island plants based on different life forms. Most studies tend to address species richness without exploring other comprehensive indicators, such as plant community abundance, relative richness, and relative abundance. Our study represents a significant advancement in this area, revealing the distinct relationships between plant richness and abundance and island area and isolation. The observed trends in species richness align with traditional island biogeography theory, while plant abundance exhibits a more complex pattern, highlighting differential responses among various plant life forms. Specifically, trees are more sensitive to spatial limitations and their growth and dispersal are more dependent on the resources and habitat space provided by larger islands, whereas shrubs exhibit stronger dispersal abilities and faster reproductive mechanisms, showing greater adaptability in resource-limited and highly isolated environments. In addition, relative richness and relative abundance also show significant differences under variations in island area and isolation. On larger islands, the relative richness and relative abundance of trees significantly increase, while shrubs show a contrasting decline. These results indicate that increasing island area favors the expansion of trees, while higher island isolation promotes the dominance of shrubs within communities, revealing the distinct responses of different plant life forms to their ecological strategies in island environments. Structural equation modeling (SEM) further indicates that island area and isolation indirectly affect plant community richness and abundance by influencing soil pH, highlighting the mediating role of soil pH as a key environmental factor. This emphasizes the critical role of soil factors in shaping the structure of island plant communities. The direct effects of island geography and climate on plant communities are not limited to the physical environment but also act indirectly through their influence on the soil, affecting plant community composition and function. This finding provides new perspectives for the conservation and management of island plant communities. Island area and isolation directly influence the composition of plant communities, and different plant life forms respond differently to these factors. Therefore, island conservation strategies should be tailored to the needs of different plant communities, taking into account the size and isolation of the islands. Additionally, future research should focus on how island plant communities respond to climate change, particularly the regulation of key soil factors such as soil pH, to further optimize the management of island ecosystems and promote plant community diversity. Long-term monitoring and comparative studies across islands will help to better understand the complex relationships between island geography, soil properties, and plant community composition, providing a more scientific basis for island conservation.

## Data Availability

The raw data supporting the conclusions of this article will be made available by the authors, without undue reservation.
