# Supplementary material for: Distribution of different plant life forms on tropical islands: patterns and underlying mechanisms
Source: Front Plant Sci. 2025 Apr 2;16:1566156. doi: 10.3389/fpls.2025.1566156 (PMC12000092; doi:10.3389/fpls.2025.1566156)

**Distribution of different plant life forms on Tropical Islands: Patterns and Underlying Mechanisms**

**Chengfeng Yang^1^, Jingyan Zhao^1^, Shengzhuo Huang^2^, Shurong Zhou^3^, Yikang Cheng^3*^**

^1^Key Laboratory of Genetics and Germplasm Innovation of Tropical Special Forest Trees and Ornamental Plants, Ministry of Education, School of Tropical Agriculture and Forestry, Hainan University, Haikou, China

^2^Hainan Key Laboratory for Research and Development of Natural Products from Li Folk Medicine, Institute of Tropical Bioscience and Biotechnology, Chinese Academy of Agricultural Sciences, Haikou 571101, China

^3^School of Ecology, Hainan University, Haikou, PR China

ORCID information:

Yikang Cheng: [https://orcid.org/0000-0003-4942-3904](https://orcid.org/0000-0003-4942-3904%20)

* Corresponding author:

Yikang Cheng: [ykcheng@hainanu.edu.cn](mailto:ykcheng@hainanu.edu.cn)

**Table S1** The loadings, proportion explained, and cumulative proportion of the first [three](http://www.youdao.com/w/three/#keyfrom=E2Ctranslation) principal components for the principal component analysis (PCA) of climate factors' dimensionality reduction and soil nutrients.

| **Variables** | **PC1** | **PC2** | **PC3** |
| --- | --- | --- | --- |
| **Climate variables** |  | |  |
| Annual mean temperature | 0.428 | 0.790 | 0.439 |
| Annual precipitation | -0.711 | -0.006 | 0.703 |
| Annual mean wind | -0.558 | 0.613 | -0.559 |
| **Proportion explained** | **0.560** | 0.339 | 0.101 |
| **Cumulative proportion** | **0.560** | 0.899 | 1.000 |
| **Soil nutrients** |  | | |
| TN | -0.658 | 0.114 | 0.233 |
| TP | -0.373 | -0.574 | -0.729 |
| TK | -0.037 | 0.793 | -0.607 |
| SOC | -0.653 | 0.167 | 0.216 |
| **Proportion explained** | **0.547** | 0.321 | 0.128 |
| **Cumulative proportion** | **0.547** | 0.868 | 0.996 |

**Table S2** The initial models and the final best-fitting regression models for the species richness and abundance of all plants, as well as the relative species richness and relative abundance of each life form

| Groups | Initial/Final models | Response variables | Models | AIC | Fixed r2 | Full r2 |
| --- | --- | --- | --- | --- | --- | --- |
| Total | initial models | richness | Logarea+Logisolation+PCclimate+PCsoil+pH | 538.459 | 0.83 | 0.87 |
|  | final models | richness | Logarea+Logisolation+PCclimate+pH | **530.666** | 0.84 | 0.87 |
|  | initial models | abundance | Logarea+Logisolation+PCclimate+PCsoil+pH | 556.974 | 0.19 | 0.38 |
|  | final models | abundance | PCclimate+pH | **537.258** | 0.18 | 0.37 |
| Tree | initial models | richness | Logarea+Logisolation+PCclimate+PCsoil+pH | 634.087 | 0.78 | 0.84 |
|  | final models | Richness | Logarea+Logisolation+PCclimate+pH | **627.196** | 0.78 | 0.84 |
|  | initial models | abundance | Logarea+Logisolation+PCclimate+PCsoil+pH | 756.364 | 0.45 | 0.63 |
|  | final models | abundance | Logarea+Logisolation+PCclimate+pH | **751.045** | 0.45 | 0.64 |
|  | initial models | [relative](http://www.youdao.com/w/relative%20abundance/#keyfrom=E2Ctranslation) Richness | Logarea+Logisolation+PCclimate+PCsoil+pH | 523.068 | 0.34 | 0.56 |
|  | final models | [relative](http://www.youdao.com/w/relative%20abundance/#keyfrom=E2Ctranslation) Richness | Logarea+Logisolation+PCclimate+pH | **514.474** | 0.35 | 0.56 |
|  | initial models | [relative](http://www.youdao.com/w/relative%20abundance/#keyfrom=E2Ctranslation) Abundance | Logarea+Logisolation+PCclimate+PCsoil+pH | 570.833 | 0.23 | 0.48 |
|  | final models | [relative](http://www.youdao.com/w/relative%20abundance/#keyfrom=E2Ctranslation) Abundance | Logarea+PCclimate+pH | **556.939** | 0.23 | 0.47 |
| Shrub | initial models | richness | Logarea+Logisolation+PCclimate+PCsoil+pH | 601.453 | 0.63 | 0.70 |
|  | final models | richness | Logarea+Logisolation | **582.907** | 0.63 | 0.70 |
|  | initial models | abundance | Logarea+Logisolation+PCclimate+PCsoil+pH | 690.531 | 0.04 | 0.32 |
|  | final models | abundance | Logarea+Logisolation | **673.707** | 0.02 | 0.32 |
|  | initial models | [relative](http://www.youdao.com/w/relative%20abundance/#keyfrom=E2Ctranslation) Richness | Logarea+Logisolation+PCclimate+PCsoil+pH | 523.068 | 0.34 | 0.56 |
|  | final models | [relative](http://www.youdao.com/w/relative%20abundance/#keyfrom=E2Ctranslation) Richness | Logarea+Logisolation+PCclimate+pH | **514.474** | 0.35 | 0.56 |
|  | initial models | [relative](http://www.youdao.com/w/relative%20abundance/#keyfrom=E2Ctranslation) Abundance | Logarea+Logisolation+PCclimate+PCsoil+pH | 570.833 | 0.23 | 0.48 |
|  | final models | [relative](http://www.youdao.com/w/relative%20abundance/#keyfrom=E2Ctranslation) Abundance | Logarea+PCclimate+pH | **556.939** | 0.23 | 0.47 |

**Figure S1.** The relationship between Island area and the number of plots


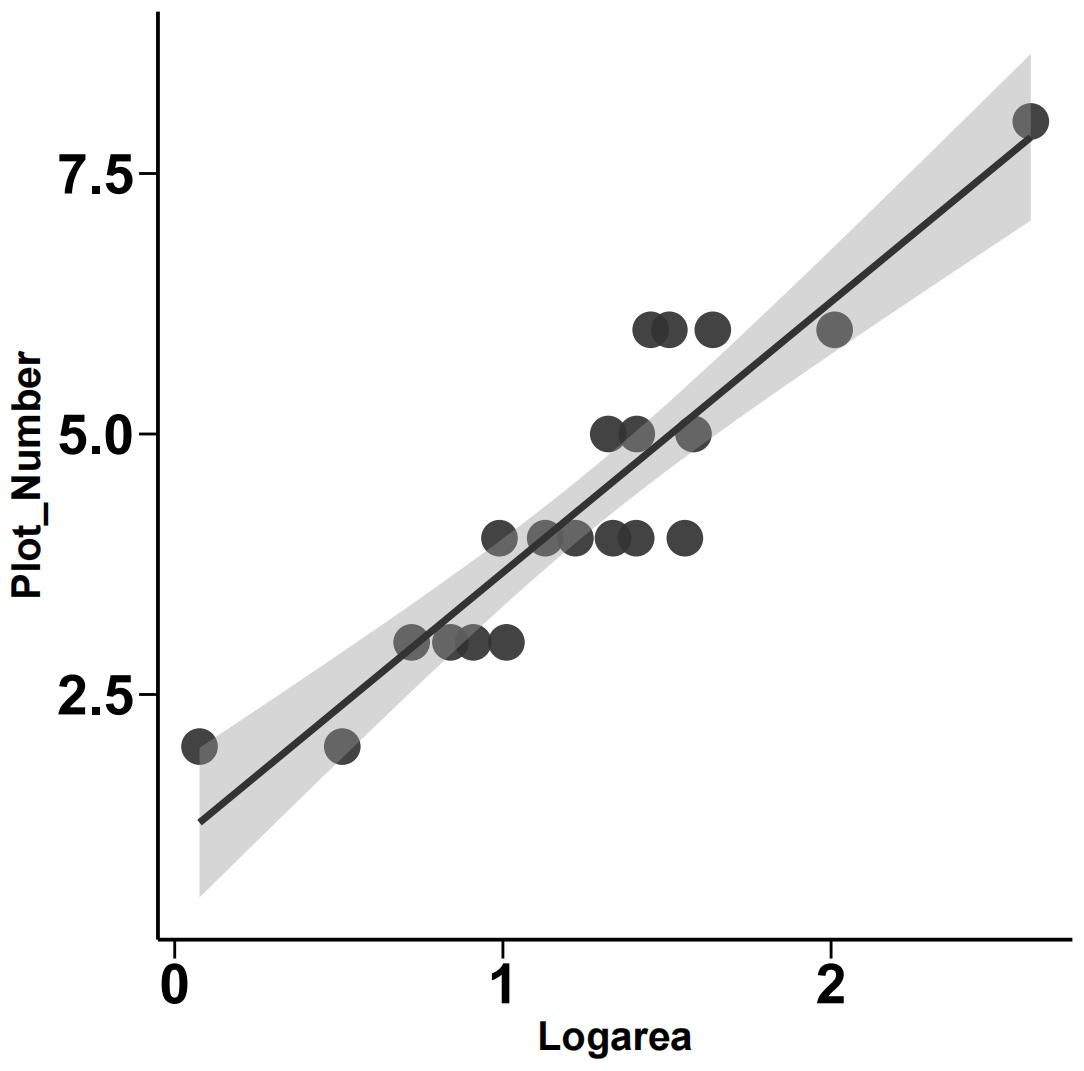


**Figure S2.** The final results of the Structural Equation Model (SEM) reveal the direct effects of island area and isolation on (Figure a) Relative richness of shrub, and (Figure b) Relative abundance of shrub, as well as their indirect effects mediated by environmental factors such as climate, soil nutrients, and pH. Red and black arrows are the standardized path coefficient variables, representing significant positive and negative pathways respectively, with the thickness of the line proportional to the strength of the path coefficient. R^2^ is the marginal value that represents the proportion of variance explained for each fixed variable in the model. The symbols denote statistical significance (***P < 0.001; **P < 0.01; *P < 0.05; ^P < 0.1).


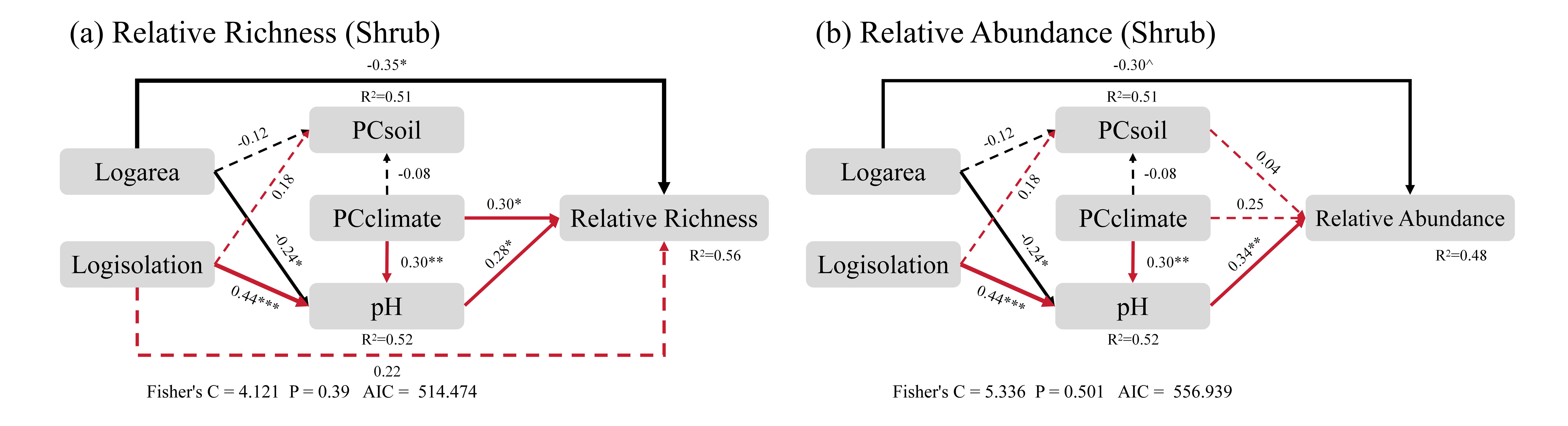

Supplement: Supplementary file 1 [file DataSheet1.docx]
